# Evaluation of the diagnostic performance of YiDiXie™ tests in urothelial carcinoma

**DOI:** 10.1186/s40001-025-03369-7

**Published:** 2025-11-12

**Authors:** Huimei Zhou, Shengjie Lin, Chen Sun, Zhenjian Ge, Wenkang Chen, Yingqi Li, Xionghui Wu, Yongqing Lai, Zhengping Zhao

**Affiliations:** 1 Department of Urology, Peking University Shenzhen Hospital, The Fifth Clinical Medical College of Anhui Medical University, 1120 Lianhua Road, Shenzhen, 518036 Guangdong China; 2 Institute of Urology, Shenzhen Peking University-The Hong Kong University of Science and Technology Medical Center, Shenzhen, 518036 China; 3 Shenzhen Clinical Research Center for Urology and Nephrology, Shenzhen, 518036 China; 4 The Fifth Clinical Medical College of Anhui Medical University, Hefei, China; 5 PKU-Shenzhen Clinical Institute of Shantou University Medical College, Shenzhen, China; 6 Shenzhen University Health Science Center, Shenzhen, China

**Keywords:** Urothelial carcinoma, miRNA biomarkers kit, Serum, Tumor

## Abstract

**Background:**

Computed tomography (CT) is widely used to the diagnosis of upper urinary tract epithelial tumors, but the relatively inaccurate results may affect subsequent treatment. Cystoscopy is widely used for preoperative diagnosis of bladder tumors and postoperative review of urothelial carcinoma, but its invasiveness and corresponding complications are feared by patients. There is an urgent need to find a convenient, cost-effective and non-invasive diagnostic method to reduce the false-positive and false-negative rates of urinary tract CT, and to replace cystoscopy in the preoperative diagnosis of bladder tumors or postoperative monitoring the bladder for recurrent of urothelial carcinoma. The study aimed to evaluate the diagnostic value of YiDiXie™-30, YiDiXie™-32, and YiDiXie™-48 in urothelial cancer.

**Materials and methods:**

439 study subjects were finally included in this study (malignant group, *n* = 292, benign group, *n* = 90; relapse group, *n* = 22, relapse-free group, *n* = 35). The remaining serum samples from the subjects were collected and tested by applying the YiDiXie™ all-cancer detection kit to evaluate the sensitivity and specificity, positive likelihood ratio, and negative likelihood ratio of YiDiXie™-30, YiDiXie™-32, and YiDiXie™-48. Receiver-operating characteristic curves’ analyses were included in this study.

**Results:**

The sensitivity, specificity, positive likelihood ratio, and negative likelihood ratio of YiDiXie™-30 were 74.7%, 92.2%, 9.6, and 0.27, respectively. The sensitivity, specificity, positive likelihood ratio, and negative likelihood ratio of YiDiXie™-32 were 86.6%, 84.4%, 5.6, and 0.16, respectively. The sensitivity, specificity, positive likelihood ratio, and negative likelihood ratio were 96.2%, 65.6%, 2.8, and 0.06, respectively. Further studies revealed that YiDiXie™-30, YiDiXie™-32, and YiDiXie™-48 had similar results in patients with positive or negative urothelial CT. The negative likelihood ratios of YiDiXie™-30, YiDiXie™-32, and YiDiXie™-48 with ultrasound concurrently in the relapse and relapse-free groups were 0.05, 0.05, and 0. In the malignant and benign groups with diameters < 2.0 cm negative likelihood ratios of YiDiXie™-30.

**Conclusion:**

YiDiXie™ tests showed considerable validity in the diagnosis of urothelial carcinoma with significantly different levels of sensitivity and specificity. They have significant diagnostic value in urothelial carcinoma, improving the diagnosis of upper urinary tract epithelial tumors and potentially providing an alternative to cystoscopy in combination with CT or ultrasound.

*Clinical study registration number *ChiCTR2200066840.

## Introduction

Urothelial carcinoma is one of the common malignant tumors [1, 2]. 90% of urothelial carcinomas are bladder urothelial carcinomas, which constitutes 49.1% of the total new cases in the urinary system [3]. In 2020, there were approximately 573,000 new cases and 213,000 deaths due to bladder cancer alone [4]. In 2022, there were approximately 614,000 new cases and 220,000 deaths [5]. Currently, surgery remains the most important treatment modality for urothelial carcinoma [6–8]. Since 60% of patients with upper urothelial carcinoma have invasive cancer at the time of diagnosis and about 9% have metastasis, the prognosis of urothelial carcinoma is very poor [6, 9]; while the 5-year overall survival rate of muscle-invasive bladder cancer patients is only 50%, and that of metastatic bladder cancer is even as low as 5% [10, 11]. Additionally, the medical expenses of bladder cancer patients are the highest among all malignant tumors [12]. Therefore, urothelial carcinoma seriously threatens human health and brings a heavy economic burden.

Computed tomography (CT) is widely used in the diagnosis of upper urothelial tumors [1, 6–8, 13, 14]; a relatively inaccurate result may affect subsequent treatment. On the one hand, CT can yield a large number of false-positive results, with a false-positive rate of 5–49% [1, 15]. When CT is positive, patients will be subjected to endoscopic examinations and other invasive procedures [1, 7, 8], which may lead to other complications for the patients [16]. On the other hand, CT can produce a large number of false-negative results, with a false-negative rate of 7.0–11.0% [1], and the false-positive rate of lesions smaller than 3 mm can reach approximately 60% [15]. When CT is negative, patients usually undergo observation and regular follow-ups, which increases unnecessary costs and the risk of malignant tumor progression [17–21]. Therefore, it is urgent to find a convenient, economical and non-invasive diagnostic method to reduce the false-positive and false-negative rates of upper urinary tract CT.

When bladder tumors are detected by imaging examinations such as physical examination or outpatient B-ultrasound, bladder endoscopy is usually performed in the outpatient department [13, 14]. Bladder endoscopy is the gold standard for diagnosing urothelial tumors and can accurately detect the number, size, shape, location, growth pattern, and surrounding bladder mucosa condition of bladder tumors [22]. Since benign bladder tumors are all treated by transurethral resection of bladder tumor (TURBT) [7, 8, 13, 14], if the benign and malignant nature of bladder tumors can be accurately determined in outpatient settings, the cystoscopy for bladder benign tumors can be avoided. Moreover, the sensitivity of CT in diagnosing bladder cancer is approximately 86.0–96.1%, and the specificity is approximately 83–92% [23–25]. Therefore, if a convenient, cost-effective and non-invasive diagnostic method can be found and combined with CT, it is expected to replace the outpatient cystoscopy for bladder tumors.

Postoperative urothelial carcinoma often requires multiple re-examinations of cystoscopy [7, 8, 13, 14]. However, its invasiveness and the corresponding complications often cause patients to be fearful and reluctant to undergo them. Patients often avoid regular postoperative cystoscopy re-examination. Patients either endure the painful examination process or fail to diagnose recurrence in time, thereby delaying treatment. Therefore, there is an urgent need to find a convenient, economical, and non-invasive diagnostic method to replace cystoscopy for the re-examination of urothelial carcinoma.

Based on the detection of novel tumor markers in serum, Shenzhen KeRuiDa Health Technology Co., Ltd. has developed an in vitro diagnostic test product YiDiXie™ all-cancer test (hereinafter referred to as “YiDiXie™ test”), which includes three products with distinct performance: YiDiXie™-30, YiDiXie™-32, and YiDiXie™-48. A single YiDiXie™ tests only requires 20 μL of serum to detect various types of cancers, including urothelial carcinoma.

Based on the above analysis, we speculate that YiDiXie™ tests can not only improve the diagnosis of upper urinary tract urothelial tumors but also be combined with CT or B-ultrasound as an alternative to cystoscopy probably. The purpose of this study is to evaluate the diagnostic value of YiDiXie™-30, YiDiXie™-32, and YiDiXie™-48 in urothelial carcinoma.

## Materials and methods

### Research design

This work is part of the ''Evaluation of the auxiliary diagnostic value of YiDiXie™ test in various tumors" sub-study of the SZ-PILOT study (ChiCTR2200066840).

The SZ-PILOT study (ChiCTR2200066840) is a single-center, prospective, observational study. The subjects who were admitted to the hospital or underwent physical examination and signed the informed consent form for donating the remaining samples were included. 0.5 mL of their remaining serum samples were collected for this study.

This study adopted a blinding method. The experimenters conducting the "YiDiXie™ test" and the technicians at the KeRuiDa Laboratory who determined the results of the "YiDiXie™ test" were all unaware of the clinical data of the research subjects. The clinical experts evaluating the clinical data of the subjects were also unaware of the test results of the "YiDiXie™ test".

This study was approved by the Ethics Committee of Peking University Shenzhen Hospital and was implemented in accordance with the Good Clinical Practice for Drug Trials of the International Conference on Harmonization and the Helsinki Declaration.

### Subjects

Three groups of subjects were enrolled separately, and all eligible subjects who met the inclusion criteria were continuously included.

This study initially included hospitalized patients with "suspected (solid or hematological) malignant tumors" who signed the informed consent form for donating the remaining samples. Recurrent subjects with a postoperative pathological diagnosis of "malignant tumor" were included in the "postoperative relapse group". Subjects with a postoperative pathological diagnosis of "malignant tumor" were included in the "malignant group", and subjects with a postoperative pathological diagnosis of "benign tumor" were included in the "benign group". Subjects with ambiguous pathological results regarding malignancy were excluded from this study.

Subjects in the "postoperative relapse-free group" initially included healthy individuals who had a history of urothelial carcinoma and signed the informed consent form for donating the remaining samples at the Health Management Center or Special Clinic of Peking University Shenzhen Hospital. Those with a definite diagnosis of malignant tumor or suspected malignant tumor but undiagnosed in their bodies did not meet the inclusion criteria for the postoperative no-recurrence group.

Subjects with unsatisfactory serum sample quality detection before YiDiXie™ tests were excluded from this study.

### Sample collection and processing

The serum samples used in this study were derived from the remaining serum after normal diagnosis and treatment, and no additional blood collection was required. Approximately 0.5 mL of serum samples were collected from the remaining serum of the subjects in the medical laboratory department and stored at − 80 ℃ for subsequent YiDiXie™ tests.

### YiDiXie™ tests

The YiDiXie™ tests are conducted using the YiDiXie™ all-cancer detection kit. This kit was developed and produced by Shenzhen KeRuiDa Health Technology Co., Ltd. and is an in vitro diagnostic kit for use with fluorescence quantitative PCR instruments. It determines whether cancer is present in the subject's body by detecting the expression levels of dozens of miRNA biomarkers in the serum. It predefines appropriate thresholds for each miRNA biomarker to ensure high specificity for each biomarker. Through a parallel test mode, it integrates these independent tests to significantly increase the sensitivity and maintaining high specificity for broad-spectrum cancers.

The YiDiXie™ tests include three distinct detection products: YiDiXie™-30, YiDiXie™-32, and YiDiXie™-48. Among them, YiDiXie™-32 balances sensitivity and specificity; YiDiXie™-48 significantly increases the number of miRNA tests to achieve extremely high sensitivity for all clinical stages of all malignant tumor types; YiDiXie™-30 significantly raises the diagnostic threshold for individual miRNA tests to achieve extremely high specificity for all malignant tumor types.

The YiDiXie™ tests are performed according to the instructions of the YiDiXie™ all-cancer detection kit. After mixing 20 μl of nucleic acid extraction solution and 20 μl of serum, it was incubated at 50 ℃ for 20 min, 95 ℃ for 5 min, and then centrifuged at 13,000 rpm for 5 min at 4 ℃. The supernatant is the crude nucleic acid extract. After mixing 8 μl of crude nucleic acid extract and 12 μl of reverse transcription reaction solution, it was incubated at 37 ℃ for 30 min, 42 ℃ for 30 min, heat at 75 ℃ for 5 min, and place on ice for 2 min. The cDNA was diluted 20 times for backup. Mixed 4 μl of the cDNA dilution solution and 6 μl of the amplification detection solution and then perform the RT-qPCR reaction program. The RT-qPCR running procedure is set as follows: 95 ℃ for 2 min, and then 40 cycles of 95 ℃ for 10 s, 60 ℃ for 30 s, and 70 ℃ for 30 s.

The original test results are analyzed by laboratory technicians from Shenzhen KeRuiDa Health Technology Co., Ltd. and are judged as "positive" or "negative" for the YiDiXie™ tests.

### CT or B-ultrasound diagnosis

The test results are determined as "positive" or "negative" based on the diagnosis conclusion of CT or B-ultrasound. Expressions, such as "positive", "relatively positive", or "tending towards malignancy", are judged as "positive". Expressions, such as "positive", "relatively positive", or "tending towards benign diseases", or expressions such as "ambiguous diagnosis of benign and malignant", are judged as "negative".

### Collection of clinical data

The clinical, pathological, laboratory, and imaging data in this study were all extracted from the hospital medical records or physical examination reports of the subjects. The assessment was completed according to the AJCC staging manual (8th edition) [26].

### Statistical analysis

For demography and baseline characteristics, descriptive statistics are reported. For classified variable, the number and percentage of subjects in each category are calculated. For continuous variables, the total number of subjects (*n*), mean value, standard deviation (SD) or standard error (SE), median, first quartile (Q1), third quartile (Q3), minimum value, and maximum value are calculated. The 95% confidence intervals (CI) of multiple indicators are calculated using the Wilson (score) method [27]. Receiver-operating characteristic (ROC) curves analyses were plotted and area under curve (AUC) was calculated.

## Result

### Subject conditions

A total of 439 participants were enrolled in this study, and stratified into four cohorts: primary malignant (*n* = 292), benign (*n* = 90), postoperative relapse (*n* = 22), and relapse-free (*n* = 35) groups. Table 1 lists the demographic and clinical characteristics of the 439 research subjects. The demographic and clinical characteristics of the two groups of research subjects were comparable (Table 1). The average (standard deviation) age was 61.4 (13.61) years, and 26.7% (117/439) were female.

**Table 1:** Participants’ demographic and clinical manifestation

	Primary				Post-op^a^				Total (*N* = 439)	
	Malignant (*n* = 292)		Benign (*n* = 90)		Relapse (*n* = 22)		Relapse-free (*n* = 35)			
Age, years										
Mean (SD)	64.1	(11.63)	52.0	(15.79)	66.7	(8.11)	49.9	(9.94)	61.4	(13.61)
Median (Q1,Q3)	65	(58, 72)	47	(38, 65)	64	(62, 72)	52	(41, 57)	64	(55, 71)
Min, max	21,	89	18,	82	48,	81	31,	69	18,	89
Age, group, *n* (%)										
< 50	28	(9.6)	42	(46.7)	1	(4.5)	14	(40.0)	85	(19.4)
≥ 50	264	(90.4)	48	(53.3)	21	(95.5)	21	(60.0)	354	(80.6)
< 65	141	(48.3)	66	(73.3)	11	(50.0)	34	(97.1)	252	(57.4)
≥ 65	151	(51.7)	24	(26.7)	11	(50.0)	1	(2.9)	187	(42.6)
Sex, *n* (%)										
Female	69	(23.6)	30	(33.3)	4	(18.2)	14	(40.0)	117	(26.7)
Male	223	(76.4)	60	(66.7)	18	(81.8)	21	(60.0)	322	(73.3)
Body mass index (kg/m^2^)										
* n*	214		62		15		32		323	
Mean (SD)	23.6	(3.26)	23.7	(3.40)	23.4	(1.29)	24.6	(3.54)	23.7	(3.28)
Median (Q1,Q3)	23.5	(21.5, 26.1)	23.7	(21.8, 25.3)	23.5	(22.5, 24.2)	23.4	(22.0, 27.0)	23.7	(21.6, 26.0)
Min, max	15.8	34.1	16.8	31.5	20.3	25.4	17.8	31.8	15.8	34.1
Body mass index category, *n* (%)										
Underweight	14	(4.8)	5	(5.6)	0	(0)	1	(2.9)	20	(4.6)
Benign	100	(34.2)	27	(30.0)	9	(40.9)	16	(45.7)	152	(34.6)
Overweight	78	(26.7)	24	(26.7)	6	(27.3)	9	(25.7)	117	(26.7)
Obese	22	(7.5)	6	(6.7)	0	(0)	6	(17.1)	34	(7.7)
Missing	78	(26.7)	28	(31.1)	7	(31.8)	3	(8.6)	116	(26.4)
Tumor location										
Bladder	245	(83.9)	88	(97.8)	22	(100)			355	(87.9)
Ureter	16	(5.5)	2	(2.2)	0	(0)			18	(4.5)
Renal pelvis	31	(10.6)	0	(0)	0	(0)			31	(7.7)
Tumor Size (cm)										
< 1.0	27	(9.2)	21	(23.3)	2	(9.1)			50	(12.4)
[1.0,1.5)	33	(11.3)	25	(27.8)	5	(22.7)			63	(15.6)
[1.5,2.0)	46	(15.8)	15	(16.7)	4	(18.2)			65	(16.1)
≥ 2.0	186	(63.7)	29	(32.2)	11	(50.0)			226	(55.9)
AJCC clinical stage										
Stage 0	147	(50.3)			8	(36.4)			155	(49.4)
Stage I	66	(22.6)			6	(27.3)			72	(22.9)
Stage II	25	(8.6)			3	(13.6)			28	(8.9)
Stage III	14	(4.8)			3	(13.6)			17	(5.4)
Stage IV	25	(8.6)			1	(4.5)			26	(8.3)
Missing	15	(5.1)			1	(4.5)			16	(5.1)

Q1,Q3, first quartile, third quartile; SD, standard deviation

^a^urothelial carcinoma postoperative group

### Diagnostic performance of YiDiXie™ tests in urothelial tumors

As shown in Table 2, the sensitivity, specificity, positive likelihood ratio, and negative likelihood ratio of YiDiXie™-30 were 74.7% (95% CI 69.2–79.5%), 92.2% (95% CI 84.1–96.5%), 9.6 (95% CI 4.7–19.6), and 0.27 (95% CI 0.23–0.34), respectively. The sensitivity of YiDiXie™-30 increased with the increase of the clinical stage.

**Table 2:** Performance of YiDiXie™ tests in malignant and benign groups

	*N*^a^	*P*^b^	Sensitivity % (95% CI)^e^	*N*^c^	Ne^d^	Specificity % (95% CI)^f^	LR + (95% CI)^g^	LR–(95% CI)^h^
YiDiXie™-30								
All	292	218	74.7% (69.2–79.5%)	90	83	92.2% (84.1–96.5%)	9.6 (4.7–19.6)	0.27 (0.23–0.34)
Stage 0	147	96	65.3% (57.0–72.8%)				8.4 (4.1–17.3)	0.38 (0.30–0.47)
Stage I	66	48	72.7% (60.2–82.6%)				9.4 (4.5–19.3)	0.30 (0.20–0.44)
Stage II	25	22	88.0% (67.7–96.8%)				11.3 (5.5–23.4)	0.13 (0.04–0.38)
Stage III	14	13	92.9% (64.2–99.6%)				11.9 (5.8–24.7)	0.08 (0.01–0.51)
Stage IV	25	25	100% (83.4–100%)				12.9 (6.3–26.2)	0 (0–NaN^i^)
Missing	15	14	93.3% (66.0–99.7%)				12.0 (5.8–24.8)	0.07 (0.01–0.48)
YiDiXie™-32								
All	292	253	86.6% (82.1–90.2%)	90	76	84.4% (74.9–90.9%)	5.6 (3.4–9.0)	0.16 (0.12–0.21)
Stage 0	147	122	83.0% (75.7–88.5%)				5.3 (3.3–8.7)	0.20 (0.14–0.29)
Stage I	66	57	86.4% (75.2–93.2%)				5.6 (3.4–9.1)	0.16 (0.09–0.30)
Stage II	25	22	88.0% (67.7–96.8%)				5.7 (3.4–9.4)	0.14 (0.05–0.41)
Stage III	14	13	92.9% (64.2–99.6%)				6.0 (3.6–9.9)	0.08 (0.01–0.56)
Stage IV	25	25	100% (83.4–100%)				6.4 (4.0–10.4)	0 (0–NaN^i^)
Missing	15	14	93.3% (66.0–99.7%)				6.0 (3.6–9.9)	0.08 (0.01–0.53)
YiDiXie™-48								
All	292	281	96.2% (93.2–98.0%)	90	59	65.6% (54.7–75.1%)	2.8 (2.1–3.7)	0.06 (0.03–0.10)
Stage 0	147	137	93.2% (87.5–96.5%)				2.7 (2.0–3.6)	0.10 (0.06–0.19)
Stage I	66	65	98.5% (90.7–100%)				2.9 (2.1–3.8)	0.02 (0–0.16)
Stage II	25	25	100% (83.4–100%)				2.9 (2.2–3.9)	0 (0–NaN^i^)
Stage III	14	14	100% (73.2–100%)				2.9 (2.2–3.9)	0 (0–NaN^i^)
Stage IV	25	25	100% (83.4–100%)				2.9 (2.2–3.9)	0 (0–NaN^i^)
Missing	15	15	100% (74.7–100%)				2.9 (2.2–3.9)	0 (0–NaN^i^)

CI, confidence interval. *N*^a^, total number of malignant. *P*^b^, positive. *N*^c^, total number of benign. Ne^d^, test negative. LR + , positive likelihood ratio. LR-, negative likelihood ratio. ^e,f,g,h^, two-sided 95% Wilson confidence intervals were calculated. ^i^, the calculation cannot be performed

The sensitivity, specificity, positive likelihood ratio, and negative likelihood ratio of YiDiXie™-32 were 86.6% (95% CI 82.1–90.2%), 84.4% (95% CI 74.9–90.9%), 5.6 (95% CI 3.4–9.0), and 0.16 (95% CI 0.12–0.21), respectively. The sensitivity of YiDiXie™-32 increased with the increase of the clinical stage.

The sensitivity, specificity, positive likelihood ratio, and negative likelihood ratio of YiDiXie™-48 were 96.2% (95% CI 93.2–98.0%), 65.6% (95% CI 54.7–75.1%), 2.8 (95% CI 2.1–3.7), and 0.06 (95% CI 0.03–0.10), respectively. The sensitivity of YiDiXie™-48 increased with the increase of the clinical stage.

**Fig. 1:**
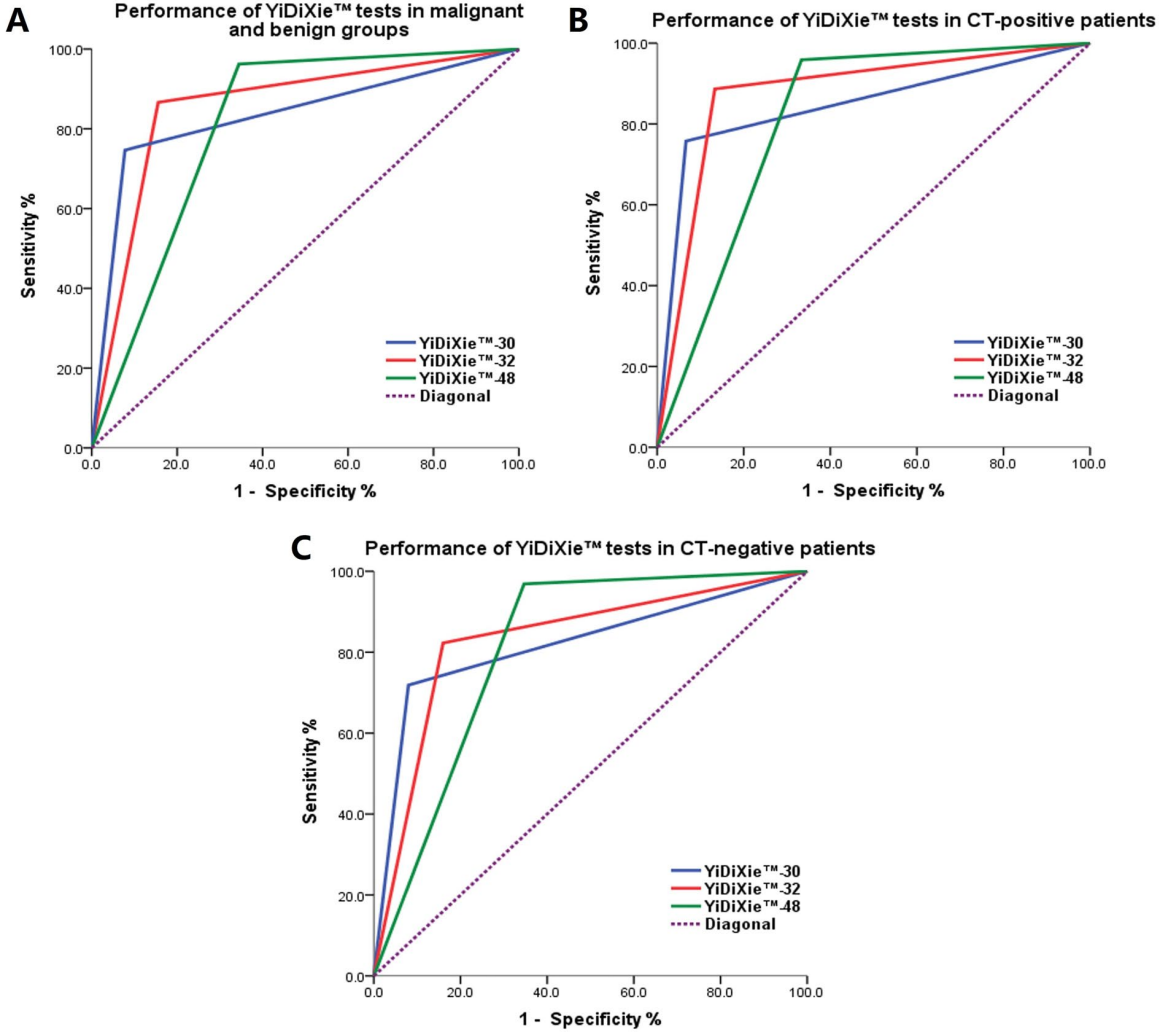
ROC curves of YiDiXie™ tests in malignant and benign groups and in CT-positive or negative patients. **A** Performance of YiDiXie™ tests in malignant groups (*n* = 292) and benign groups (*n* = 90). The AUC for YiDiXie™-30, YiDiXie™-32, and YiDiXie™-48 were 0.834, 0.855, and 0.809, respectively (*p* < 0.001). **B** Performance of YiDiXie™ tests in CT-positive patients (*n* = 194). The AUC for YiDiXie™-30, YiDiXie™-32, and YiDiXie™-48 were 0.846, 0.877, and 0.813, respectively (*p* < 0.001). **C** Performance of YiDiXie™ tests in CT-negative patients (*n* = 96). The AUC for YiDiXie™-30, YiDiXie™-32, and YiDiXie™-48 were 0.819, 0.831, and 0.811, respectively (*p* < 0.001)

As shown in Fig. 1A, the AUC for YiDiXie™-30, YiDiXie™-32, and YiDiXie™-48 in malignant and benign groups were 0.834, 0.855, and 0.809, respectively (*p* < 0.001).

### Diagnostic performance of YiDiXie™-30 in urothelial CT-positive and negative patients

To reduce the false-positive rate of urothelial CT, YiDiXie™-30 was applied to CT-positive patients. As shown in Table 3, the sensitivity, specificity, positive likelihood ratio, and negative likelihood ratio of YiDiXie™-30 in CT-positive patients were 75.8% (95% CI 69.0–81.5%), 93.3% (95% CI 66.0–99.7%), 11.4 (95% CI 1.7–75.6), and 0.26 (95% CI 0.20–0.34), respectively.

**Table 3:** Performance of YiDiXie™ tests in CT-positive or negative patients

	*N*^a^	*P*^b^	Sensitivity % (95% CI)^e^	*N*^c^	Ne^d^	Specificity % (95% CI)^f^	LR + (95% CI)^g^	LR–(95% CI)^h^
YiDiXie™-30 in CT-positive	194	147	75.8% (69.0–81.5%)	15	14	93.3% (66.0–99.7%)	11.4 (1.7–75.6)	0.26 (0.20–0.34)
YiDiXie™-32 in CT-positive	194	172	88.7% (83.1–92.6%)	15	13	86.7% (58.4–96.7%)	6.6 (1.8–24.2)	0.13 (0.09–0.20)
YiDiXie™-48 in CT-positive	194	186	95.9% (91.7–98.1%)	15	10	66.7% (38.7–87.0%)	2.9 (1.4–5.9)	0.06 (0.03–0.13)
YiDiXie™-30 in CT-negative	96	69	71.9% (61.6–80.3%)	75	69	92.0% (82.8–96.7%)	9.0 (4.1–19.6)	0.30 (0.22–0.42)
YiDiXie™-32 in CT-negative	96	79	82.3% (72.9–89.1%)	75	63	84.0% (73.3–91.1%)	5.1 (3.0–8.7)	0.21 (0.14–0.33)
YiDiXie™-48 in CT-negative	96	93	96.9% (90.5–99.2%)	75	49	65.3% (53.4–75.7%)	2.8 (2.0–3.8)	0.05 (0.02–0.15)

CI, confidence interval. N^a^, total number of malignant. P^b^, positive. N^c^, total number of benign. Ne^d^, test negative. LR + , positive likelihood ratio. LR-, negative likelihood ratio. ^e,f,g,h^, two-sided 95% Wilson confidence intervals were calculated

To reduce the false-negative rate of urothelial CT, YiDiXie™-30 was applied to CT-negative patients. As shown in Table 3, the sensitivity, specificity, positive likelihood ratio, and negative likelihood ratio of YiDiXie™-30 in CT-negative patients were 71.9% (95% CI 61.6–80.3%), 92.0% (95% CI 82.8–96.7%), 9.0 (95% CI 4.1–19.6), and 0.30 (95% CI 0.22–0.42), respectively.

As shown in Fig. 1B, C, the AUC for YiDiXie™-30 in CT-positive patients and in CT-negative patients were 0.846 and 0.819 (*p* < 0.001).

### Diagnostic performance of YiDiXie™-32 in urothelial CT-positive and negative patients

To reduce the false-positive rate of CT in urothelial carcinoma, YiDiXie™-32 was applied to patients with positive CT results. As shown in Table 3, the sensitivity, specificity, positive likelihood ratio, and negative likelihood ratio of YiDiXie™-32 in patients with positive CT results were 88.7% (95% CI 83.1–92.6%), 86.7% (95% CI 58.4–96.7%), 6.6 (95% CI 1.8–24.2), and 0.13 (95% CI 0.09–0.20), respectively.

To reduce the false-negative rate of CT in urothelial carcinoma, YiDiXie™-32 was applied to patients with negative CT results. As shown in Table 3, the sensitivity, specificity, positive likelihood ratio, and negative likelihood ratio of YiDiXie™-32 in patients with negative CT results were 82.3% (95% CI 72.9–89.1%), 84.0% (95% CI 73.3–91.1%), 5.1 (95% CI 3.0–8.7), and 0.21 (95% CI 0.14–0.33), respectively.

As shown in Fig. 1B, C, the AUC for YiDiXie™-32 in CT-positive patients and in CT-negative patients were 0.877 and 0.831 (*p* < 0.001).

### Diagnostic performance of YiDiXie™-48 in urothelial CT-positive and negative patients

To reduce the false-positive rate of CT in urothelial carcinoma, YiDiXie™-48 was applied to patients with positive CT results. As shown in Table 3, the sensitivity, specificity, positive likelihood ratio, and negative likelihood ratio of YiDiXie™-48 in patients with positive CT results were 95.9% (95% CI 91.7–98.1%), 66.7% (95% CI 38.7–87.0%), 2.9 (95% CI 1.4–5.9), and 0.06 (95% CI 0.03–0.13), respectively.

To reduce the false-negative rate of CT in urothelial carcinoma, YiDiXie™-48 was applied to patients with negative CT results. As shown in Table 3, the sensitivity, specificity, positive likelihood ratio, and negative likelihood ratio of YiDiXie™-48 in patients with negative CT results were 96.9% (95% CI 90.5–99.2%), 65.3% (95% CI 53.4–75.7%), 2.8 (95% CI 2.0–3.8), and 0.05 (95% CI 0.02–0.15), respectively.

As shown in Fig. 1B, C, the AUC for YiDiXie™-48 in CT-positive patients and in CT-negative patients were 0.813 and 0.811 (*p* < 0.001).

### Diagnostic performance of YiDiXie™ tests combined with CT in urinary tract epithelial tumors of the bladder

Table 4 shows the performance of CT, YiDiXie™ tests, and their combination.

**Table 4:** Performance of YiDiXie™ tests combined CT in malignant and benign groups

	*N*^a^	*P*^b^	Sensitivity % (95% CI)^e^	*N*^c^	Ne^d^	Specificity % (95% CI)^f^	LR + (95% CI)^g^	LR–(95% CI)^h^
CT								
All	290	194	66.9% (61.1–72.2%)	90	75	83.3% (73.7–90.1%)	4.0 (2.5–6.4)	0.40 (0.34–0.47)
< 2.0	106	58	54.7% (44.8–64.3%)	61	54	88.5% (77.2–94.9%)	4.8 (2.3–9.8)	0.51 (0.41–0.63)
≥ 2.0	184	136	73.9% (66.8–80.0%)	29	21	72.4% (52.5–86.6%)	2.7 (1.5–4.9)	0.36 (0.28 -0.47)
YiDiXie™-30								
All	290	216	74.5% (69.0–79.3%)	90	83	92.2% (84.1–96.5%)	9.6 (4.7–19.6)	0.28 (0.23–0.34)
< 2.0	106	68	64.2% (54.2–73.1%)	61	54	88.5% (77.2–94.9%)	5.6 (2.7–11.4)	0.40 (0.31–0.52)
≥ 2.0	184	148	80.4% (73.8–85.8%)	29	29	100% (85.4–100%)	Infinity (Infinity–NaN^i^)	0.20 (0.15–0.26)
YiDiXie™-32								
All	290	251	86.6% (82.0–90.2%)	90	76	84.4% (74.9–90.9%)	5.6 (3.4–9.0)	0.16(0.12–0.21)
< 2.0	106	88	83.0% (74.2–89.4%)	61	47	77.0% (64.2–86.5%)	3.6 (2.3–5.8)	0.22 (0.14–0.34)
≥ 2.0	184	163	88.6% (82.9–92.6%)	29	29	100% (85.4–100%)	Infinity (Infinity–NaN^i^)	0.11 (0.08–0.17)
YiDiXie™-48								
All	290	279	96.2% (93.1–98.0%)	90	59	65.6% (54.7–75.1%)	2.8 (2.1–3.7)	0.06 (0.03–0.10)
< 2.0	106	100	94.3% (87.6–97.7%)	61	39	63.9% (50.6–75.5%)	2.6 (1.9–3.7)	0.09 (0.04–0.20)
≥ 2.0	184	179	97.3% (93.4–99.0%)	29	20	69.0% (49.0–84.0%)	3.1 (1.8–5.4)	0.04 (0.02–0.10)
YiDiXie™-30 & CT								
All	290	263	90.7% (86.6–93.7%)	90	69	76.7% (66.3–84.7%)	3.9 (2.7–5.7)	0.12 (0.08–0.18)
< 2.0	106	94	88.7% (80.7–93.8%)	61	48	78.7% (66.0–87.7%)	4.2 (2.6–6.8)	0.14 (0.08–0.25)
≥ 2.0	184	169	91.8% (86.7–95.2%)	29	21	72.4% (52.5–86.6%)	3.3 (1.8–6.0)	0.11 (0.07–0.19)
YiDiXie™-32 & CT								
All	290	273	94.1% (90.6–96.4%)	90	63	70.0% (59.3–79.0%)	3.1 (2.3–4.3)	0.08 (0.05–0.13)
< 2.0	106	99	93.4% (86.4–97.1%)	61	42	68.9% (55.6–79.8%)	3.0 (2.1–4.4)	0.10 (0.05–0.20)
≥ 2.0	184	174	94.6% (90.0–97.2%)	29	21	72.4% (52.5–86.6%)	3.4 (1.9–6.2)	0.08 (0.04–0.14)
YiDiXie™-48 & CT								
All	290	287	99.0% (96.8–99.7%)	90	49	54.4% (43.6–64.9%)	2.2 (1.7–2.7)	0.02 (0.01–0.06)
< 2.0	106	104	98.1% (92.7–99.7%)	61	35	57.4% (44.1–69.7%)	2.3 (1.7–3.1)	0.03 (0.01–0.13)
≥ 2.0	184	183	99.5% (96.5–100%)	29	14	48.3% (29.9–67.1%)	1.9 (1.4–2.7)	0.01 (0–0.01)

CI, confidence interval. *N*^a^, total number of malignant. *P*^b^, positive. *N*^c^, total number of benign. Ne^d^, negative. LR + , positive likelihood ratio. LR-, negative likelihood ratio. ^e,f,g,h^, two-sided 95% Wilso n confidence intervals were calculated. ^i^, the calculation cannot be performed

To improve the sensitivity of CT in diagnosing urinary tract epithelial tumors of the bladder and avoid missed diagnosis and delayed treatment, we combined YiDiXie™ tests with CT in a parallel form. Only when both YiDiXie™ tests and CT results were negative would it be determined as "negative", and the rest would be "positive". As shown in Table 4, the sensitivity, specificity, positive likelihood ratio, and negative likelihood ratio of YiDiXie™-30 combined with CT were 90.7% (86.6–93.7%), 76.7% (66.3–84.7%), 3.9 (2.7–5.7), and 0.12 (0.08–0.18), respectively. The sensitivity, specificity, positive likelihood ratio, and negative likelihood ratio of YiDiXie™-32 combined with CT were 94.1% (90.6–96.4%), 70.0% (59.3–79.0%), 3.1 (2.3–4.3), and 0.08 (0.05–0.13), respectively. The sensitivity, specificity, positive likelihood ratio, and negative likelihood ratio of YiDiXie™-48 combined with CT were 99.0% (96.8–99.7%), 54.4% (43.6–64.9%), 2.2 (1.7–2.7), and 0.02 (0.01–0.06), respectively.

**Fig. 2:**
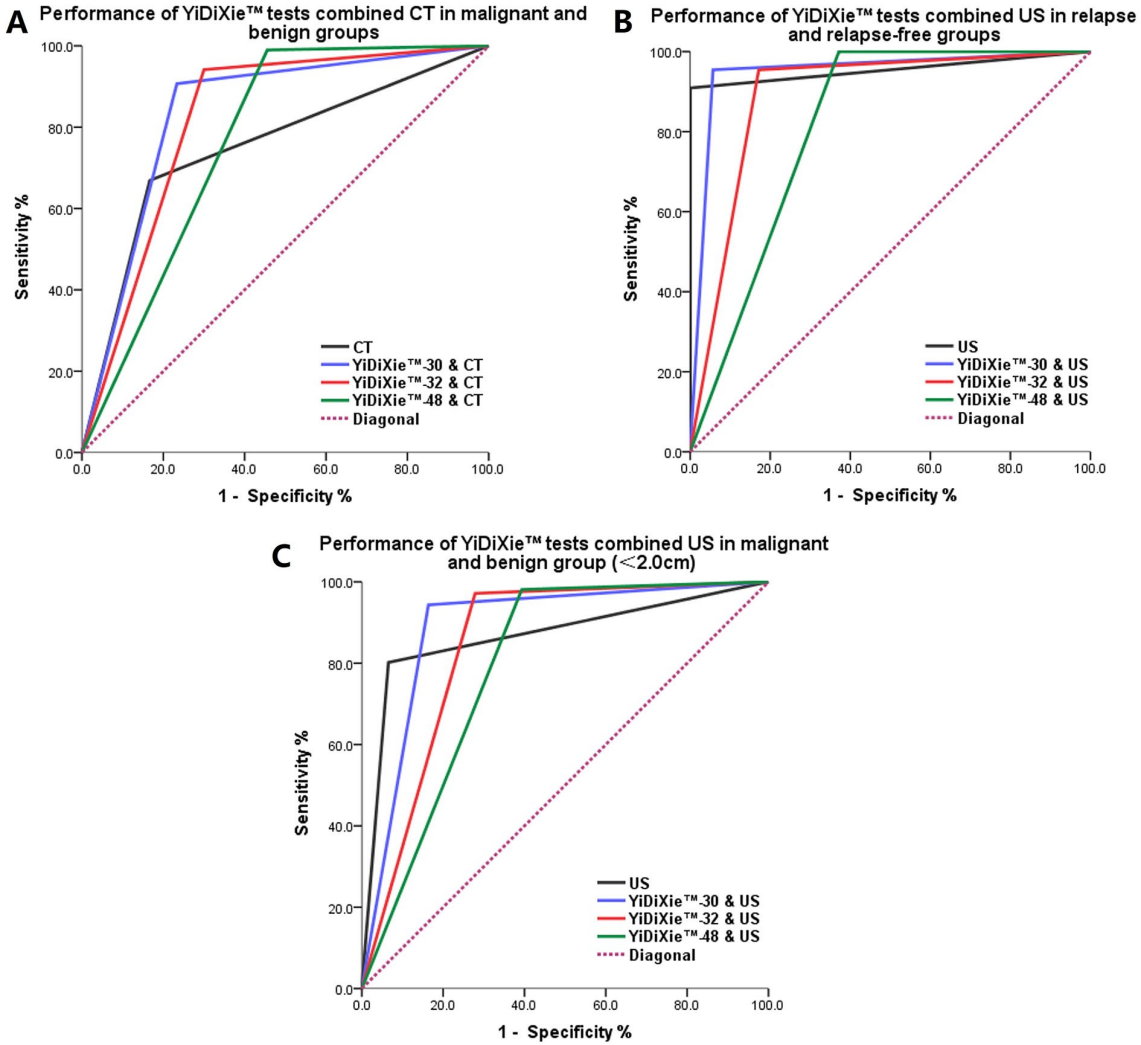
ROC curves of YiDiXie™ tests combined CT or US. **A** Performance of YiDiXie™ tests combined CT in malignant (*n* = 290) and benign groups (*n* = 90). The AUC for CT, YiDiXie™-30 & CT, YiDiXie™-32 & CT, and YiDiXie™-48 & CT were 0.751, 0.837, 0.821, and 0.767, respectively (*p* < 0.001). **B** Performance of YiDiXie™ tests combined US in relapse (*n* = 22) and relapse-free groups (*n* = 35). The AUC for US, YiDiXie™-30 & US, YiDiXie™-32 & US, and YiDiXie™-48 & US were 0.955, 0.949, 0.892 and 0.814, respectively (*p* < 0.001). **C** Performance of YiDiXie™ tests combined US in malignant (*n* = 106) and benign group (*n* = 61) (< 2.0 cm). The AUC for US, YiDiXie™-30 & US, YiDiXie™-32 & US, and YiDiXie™-48 & US were 0.868, 0.890, 0.847, and 0.794, respectively (*p* < 0.001)

As shown in Fig. 2A, the AUC for CT, YiDiXie™-30 & CT, YiDiXie™-32 & CT, YiDiXie™-48, & CT in malignant and benign groups were 0.751, 0.837, 0.821, and 0.767, respectively (*p* < 0.001).

### The diagnostic performance of YiDiXie™ tests combined with B-ultrasound in postoperative monitoring of recurrence in urothelial carcinoma.

Table 5 shows the performance of B-ultrasound, YiDiXie™ tests, and their combination in the postoperative relapse group and the relapse-free group.

**Table 5:** Performance of YiDiXie™ tests combined US in relapse and relapse-free groups

	*N*^a^	*P*^b^	Sensitivity % (95% CI)^e^	*N*^c^	Ne^d^	Specificity % (95% CI)^f^	LR + (95% CI)^g^	LR-(95% CI)^h^
US								
All	22	20	90.9% (69.4–98.4%)	35	*35*	100% (87.7–100%)	Infinity (Infinity–NaN^i^)	0.09 (0.02–0.34)
< 2.0	11	9	81.8% (47.8–96.8%)				Infinity (Infinity–NaN^i^)	0.18 (0.05–0.64)
≥ 2.0	11	11	100% (67.9–100%)				Infinity (Infinity–NaN^i^)	0 (0–NaN^i^)
YiDiXie™-30								
All	22	19	86.4% (64.0–96.4%)	35	33	94.3% (79.5–99.0%)	15.1 (3.9–58.6)	0.14 (0.05–0.42)
< 2.0	11	8	72.7% (39.3–92.7%)				12.7 (3.2–51.3)	0.29 (0.11–0.76)
≥ 2.0	11	11	100% (67.9–100%)				17.5 (4.6–67.2)	0 (0–NaN^i^)
YiDiXie™-32								
All	22	20	90.9% (69.4–98.4%)	35	29	82.9% (65.7–92.8%)	5.3 (2.5–11.1)	0.11 (0.03–0.42)
< 2.0	11	9	81.8% (47.8–96.8%)				4.8 (2.2–10.4)	0.22 (0.06–0.78)
≥ 2.0	11	11	100% (67.9–100%)				5.8 (2.8–12.1)	0 (0–NaN^i^)
YiDiXie™-48								
All	22	21	95.5% (75.1–99.8%)	35	22	62.9% (44.9–78.0%)	2.6 (1.7–4.0)	0.07 (0.01–0.51)
< 2.0	11	10	90.9% (57.1–99.5%)				2.4 (1.5–3.9)	0.14 (0.02–0.97)
≥ 2.0	11	11	100% (67.9–100%)				2.7 (1.7–4.1)	0 (0–NaN^i^)
YiDiXie™-30 & US								
All	22	21	95.5% (75.1–99.8%)	35	33	94.3% (79.5–99.0%)	16.7 (4.3–64.4)	0.05 (0–0.33)
< 2.0	11	10	90.9% (57.1–99.5%)				15.9 (4.1–61.9)	0.10 (0.01–0.63)
≥ 2.0	11	11	100% (67.9–100%)				17.5 (4.6–67.2)	0 (0–NaN^i^)
YiDiXie™-32 & US								
All	22	21	95.5% (75.1–99.8%)	35	29	82.9% (65.7–92.8%)	5.6 (2.7–11.6)	0.05 (0–0.37)
< 2.0	11	10	90.9% (57.1–99.5%)				5.3 (2.5–11.2)	0.11 (0.02–0.72)
≥ 2.0	11	11	100% (67.9–100%)				5.8 (2.8–12.1)	0 (0–NaN^i^)
YiDiXie™-48 & US								
All	22	22	100% (81.5–100%)	35	22	62.9% (44.9–78.0%)	2.7 (1.7–4.1)	0 (0–NaN^i^)
< 2.0	11	11	100% (67.9–100%)				2.7 (1.7–4.1)	0 (0–NaN^i^)
≥ 2.0	11	11	100% (67.9–100%)				2.7 (1.7–4.1)	0 (0–NaN^i^)

CI, confidence interval. *N*^a^, total number of relapse. *P*^b^, positive. *N*^c^, total number of relapse-free. Ne^d^, negative. LR + , positive likelihood ratio. LR-, negative likelihood ratio. ^e,f,g,h^, two-sided 95% Wilso n confidence intervals were calculated. ^i^, the calculation cannot be performed

To improve the sensitivity of B-ultrasound in diagnosing postoperative recurrence of urothelial carcinoma and avoid missed diagnosis and delayed treatment, we combined YiDiXie™ tests with B-ultrasound in parallel. Only when the results of YiDiXie™ tests and B-ultrasound were both negative would it be determined as "negative", and the rest would be "positive". As shown in Table 5, the sensitivity, specificity, positive likelihood ratio, and negative likelihood ratio of YiDiXie™-30 combined with B-ultrasound were 95.5% (95% CI 75.1–99.8%), 94.3% (95% CI 79.5–99.0%), 16.7 (95% CI 4.3–64.4), and 0.05 (95% CI 0–0.33). The sensitivity, specificity, positive likelihood ratio, and negative likelihood ratio of YiDiXie™-32 combined with B-ultrasound were 95.5% (95% CI 75.1–99.8%), 82.9% (95% CI 65.7–92.8%), 5.6 (95% CI 2.7–11.6), and 0.05 (95% CI 0–0.37). The sensitivity, specificity, positive likelihood ratio, and negative likelihood ratio of YiDiXie™-48 combined with B-ultrasound were 100% (95% CI 81.5–100%), 62.9% (95% CI 44.9–78.0%), 2.7 (95% CI 1.7–4.1), and 0 (95% CI 0–NaN^i^). As shown in Fig. 2B, the AUC for US, YiDiXie™-30 & US, YiDiXie™-32 & US, YiDiXie™-48, & US in relapse and relapse-free groups were 0.955, 0.949, 0.892, and 0.814, respectively (*p* < 0.001).

To further evaluate the diagnostic performance of YiDiXie™ tests combined with B-ultrasound in postoperative monitoring of recurrence in urothelial carcinoma, the performance of B-ultrasound, YiDiXie™ tests, and their combination was determined in the malignant group with diameter < 2.0 cm and the benign group (Table 6).

**Table 6:** Performance of YiDiXie™ tests combined US in malignant and benign group (< 2.0 cm)

	*N*^a^	*P*^b^	Sensitivity % (95% CI)^e^	*N*^c^	Ne^d^	Specificity % (95% CI)^f^	LR + (95% CI)^g^	LR-(95% CI)^h^
US								
All	106	85	80.2% (71.1–87.1%)	61	55	90.2% (79.1–95.9%)	8.2 (3.8–17.5)	0.22 (0.15–0.32)
< 1.0	27	20	74.1% (53.4–88.1%)	21	20	95.2% (74.1–99.8%)	15.6 (2.3–106.7)	0.27 (0.14–0.52)
[1.0,1.5)	33	28	84.8% (67.3–94.3%)	25	23	92.0% (72.5–98.6%)	10.6 (2.8–40.4)	0.16 (0.07–0.37)
[1.5,2.0)	46	37	80.4% (65.6–90.1%)	15	14	93.3% (66.0–99.7%)	12.1 (1.8–80.6)	0.21 (0.12–0.38)
YiDiXie™-30								
All	106	68	64.2% (54.2–73.1%)	61	54	88.5% (77.2–94.9%)	5.6 (2.7–11.4)	0.40 (0.31–0.52)
< 1.0	27	14	51.9% (32.4–70.8%)	21	19	90.5% (68.2–98.3%)	5.4 (1.4–21.4)	0.53 (0.36–0.79)
[1.0,1.5)	33	21	63.6% (45.1–79.0%)	25	22	88.0% (67.7–96.8%)	5.3 (1.8–15.8)	0.41 (0.26–0.66)
[1.5,2.0)	46	33	71.7% (56.3–83.5%)	15	13	86.7% (58.4–97.7%)	5.4 (1.5–19.8)	0.33 (0.20–0.53)
YiDiXie™-32								
All	106	88	83.0% (74.2–89.4%)	61	47	77.0% (64.2–86.5%)	3.6 (2.3–5.8)	0.22 (0.14–0.34)
< 1.0	27	20	74.1% (53.4–88.1%)	21	17	81.0% (57.4–93.7%)	3.9 (1.6–9.7)	0.32 (0.17–0.62)
[1.0,1.5)	33	27	81.8% (63.9–92.4%)	25	18	72.0% (50.4–87.1%)	2.9 (1.5–5.6)	0.25 (0.12–0.54)
[1.5,2.0)	46	41	89.1% (75.6–95.9%)	15	12	80.0% (51.4–94.7%)	4.5 (1.6–12.3)	0.14 (0.06–0.32)
YiDiXie™-48								
All	106	100	94.3% (87.6–97.7%)	61	39	63.9% (50.6–75.5%)	2.6 (1.9–3.7)	0.09 (0.04–0.20)
< 1.0	27	24	88.9% (69.7–97.1%)	21	15	71.4% (47.7–87.8%)	3.1 (1.6–6.2)	0.16 (0.05–0.47)
[1.0,1.5)	33	31	93.9% (78.4–98.9%)	25	14	56.0% (35.3–75.0%)	2.1 (1.4–3.4)	0.11 (0.03–0.44)
[1.5,2.0)	46	45	97.8% (87.0–99.9%)	15	10	66.7% (38.7–87.0%)	2.9 (1.4–6.0)	0.03 (0–0.24)
YiDiXie™-30 & US								
All	106	100	94.3% (87.6–97.7%)	61	53	86.9% (75.2–93.8%)	7.2 (3.8–13.7)	0.07 (0.03–0.14)
< 1.0	27	24	88.9% (69.7–97.1%)	21	20	95.2% (74.1–99.8%)	18.7 (2.7–127.0)	0.12 (0.04–0.34)
[1.0,1.5)	33	33	100% (87.0–100%)	25	20	80.0% (58.7–92.4%)	5.0 (2.3–11.0)	0 (0–NaN^i^)
[1.5,2.0)	46	43	93.5% (81.1–98.3%)	15	13	86.7% (58.4–97.7%)	7.0 (1.9–25.5)	0.08 (0.02–0.23)
YiDiXie™-32 & US								
All	106	103	97.2% (91.3–99.3%)	61	44	72.1% (59.0–82.5%)	3.5 (2.3–5.2)	0.04 (0.01–0.12)
< 1.0	27	26	96.3% (79.1–99.8%)	21	16	76.2% (52.5–90.9%)	4.0 (1.9–8.7)	0.05 (0–0.34)
[1.0,1.5)	33	33	100% (87.0–100%)	25	16	64.0% (42.6–81.3%)	2.8 (1.6–4.7)	0 (0–NaN^i^)
[1.5,2.0)	46	44	95.7% (84.0–99.2%)	15	12	80.0% (51.3–94.7%)	4.8 (1.7–13.2)	0.05 (0.01–0.22)
YiDiXie™-48 & US								
All	106	104	98.1% (92.7–99.7%)	61	37	60.7% (47.3–72.7%)	2.5 (1.8–3.4)	0.03 (0–0.13)
< 1.0	27	26	96.3% (79.1–99.8%)	21	14	66.7% (43.1–84.5%)	2.9 (1.6–5.3)	0.06 (0–0.40)
[1.0,1.5)	33	33	100% (87.0–100%)	25	13	52.0% (31.8–71.7%)	2.1 (1.4–3.1)	0 (0–NaN^i^)
[1.5,2.0)	46	45	97.8% (87.0–99.9%)	15	10	66.7% (38.7–87.0%)	2.9 (1.4–6.0)	0.03 (0–0.24)

CI, confidence interval. *N*^a^, total number of malignant. *P*^b^, positive. *N*^c^, total number of benign. Ne^d^, negative. LR + , positive likelihood ratio. LR-, negative likelihood ratio. ^e,f,g,h^, two-sided 95% Wilson confidence intervals were calculated. ^i^, the calculation cannot be performed

As shown in Table 6, the sensitivity, specificity, positive likelihood ratio, and negative likelihood ratio of YiDiXie™-30 combined with B-ultrasound were 94.3% (95% CI 87.6–97.7%), 86.9% (95% CI 75.2–93.8%), 7.2 (95% CI 3.8–13.7), and 0.07 (95% CI 0.03–0.14). The sensitivity, specificity, positive likelihood ratio, and negative likelihood ratio of YiDiXie™-32 combined with B-ultrasound were 97.2% (95% CI 91.3–99.3%), 72.1% (95% CI 59.0–82.5%), 3.5 (95% CI 2.3–5.2), and 0.04 (95% CI 0.01–0.12), respectively. The sensitivity, specificity, positive likelihood ratio, and negative likelihood ratio of YiDiXie™-48 combined with B-ultrasound were 98.1% (95% CI 92.7–99.7%), 60.7% (95% CI 47.3–72.7%), 2.5 (95% CI 1.8–3.4), and 0.03 (95% CI 0–0.13), respectively. As shown in Fig. 2C, the AUC for US, YiDiXie™-30 & US, YiDiXie™-32 & US, YiDiXie™-48, & US in malignant and benign group were 0.868, 0.890, 0.847, and 0.794, respectively (*p* < 0.001).

## Discussion

The search for non-invasive and non-destructive detection methods has always been an important direction in clinical diagnosis research. This study evaluated the value of YiDiXie™ tests in the diagnosis of urothelial carcinoma. The research results indicated that the three tests, YiDiXie™-30, YiDiXie™-32, and YiDiXie™-48, each have their own characteristics in the diagnosis of urothelial carcinoma. The sensitivity ranking of the three tests was YiDiXie™-48 > YiDiXie™-32 > YiDiXie™-30, and the specificity ranking was YiDiXie™-30 > YiDiXie™-32 > YiDiXie™-48. They can meet the different diagnostic needs in different clinical scenarios.

**Fig. 3:**
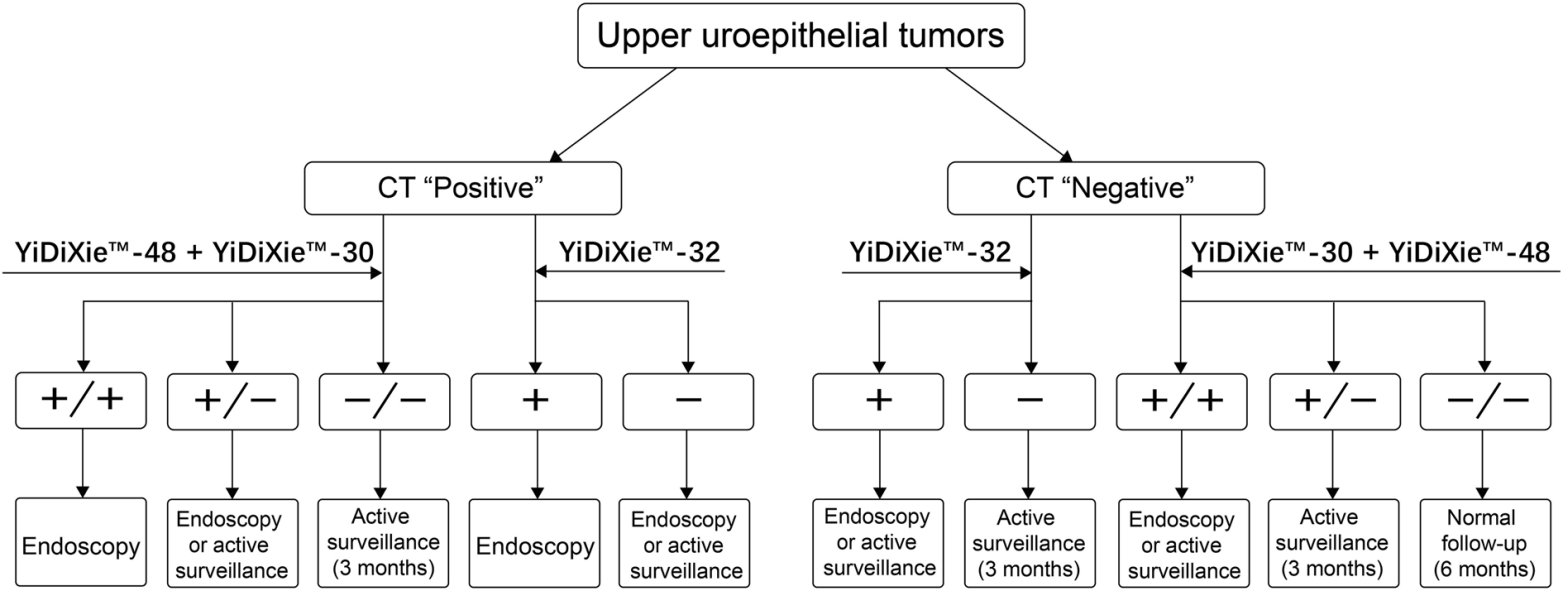
Recommended clinical pathway for YiDiXie™ tests in diagnosis of upper uroepithelial tumors

The higher the positive likelihood ratio, the more likely a diagnostic tool is to establish a diagnosis, while the lower the negative likelihood ratio, the more likely a diagnostic tool is to exclude a diagnosis [28]. The positive likelihood ratio of YiDiXie™-32 was 5.6 (95% CI 3.4–9.0), and its negative likelihood ratio was 0.16 (95% CI 0.12–0.21). In addition, YiDiXie™-32 had similar results in patients with positive or negative urothelial CT. This indicates that YiDiXie™-32 can effectively establish a malignant diagnosis and effectively rule out a malignant diagnosis. As shown in Fig. 3, YiDiXie™-32 established two diagnostic partitions in patients with positive or negative urothelial CT. Clinicians can take corresponding intervention measures based on the recommended clinical pathways, thereby improving the accuracy and timeliness of diagnosis and treatment.

A positive likelihood ratio greater than 10 means that a diagnostic tool can establish a diagnosis well, while a negative likelihood ratio less than 0.1 means that a diagnostic tool can exclude a diagnosis well [40]. The positive likelihood ratio of YiDiXie™-30 in urothelial tumors was 9.6 (95% CI 4.7–19.6), and the negative likelihood ratio of YiDiXie™-48 was 0.06 (95% CI 0.03–0.10). Both had similar results in patients with positive or negative urothelial CT. This indicates that YiDiXie™-30 can effectively establish a malignant diagnosis, while YiDiXie™-48 can effectively rule out a malignant diagnosis. As shown in Fig. 3, YiDiXie™-30 and YiDiXie™-48 established three diagnostic partitions in patients with positive or negative urothelial CT. Urologists can take corresponding intervention measures based on the clinical pathways they recommend, thereby improving the accuracy and timeliness of diagnosis and treatment.

**Fig. 4:**
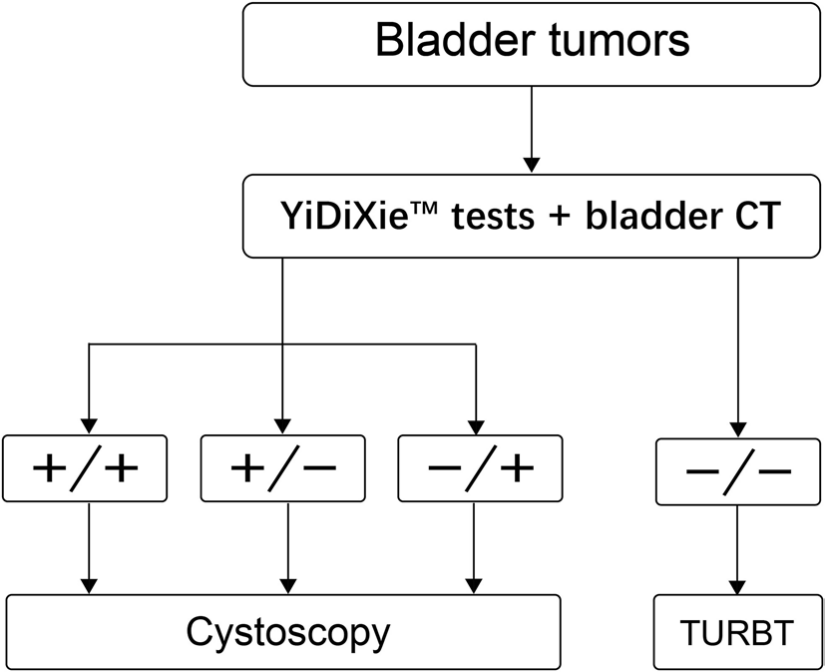
Recommended clinical pathway for YiDiXie™ tests in combination with CT as an alternative to cystoscopy in preoperative diagnosis

The negative likelihood ratios of YiDiXie™-32 or -48 in combination with CT were both less than 0.1 (Table 4); thus, these two YiDiXie™ tests in combination with CT could effectively diagnose bladder tumors and potentially offer an alternative to cystoscopy. Clinicians could take corresponding intervention measures based on the clinical pathways recommended by them (Fig. 4). The sensitivities and specificities of the YiDiXie™ tests in combination with CT were different. Clinicians could choose any one of the YiDiXie™ tests in combination with CT according to the patients' needs and the local supply of cystoscopes.

**Fig. 5:**
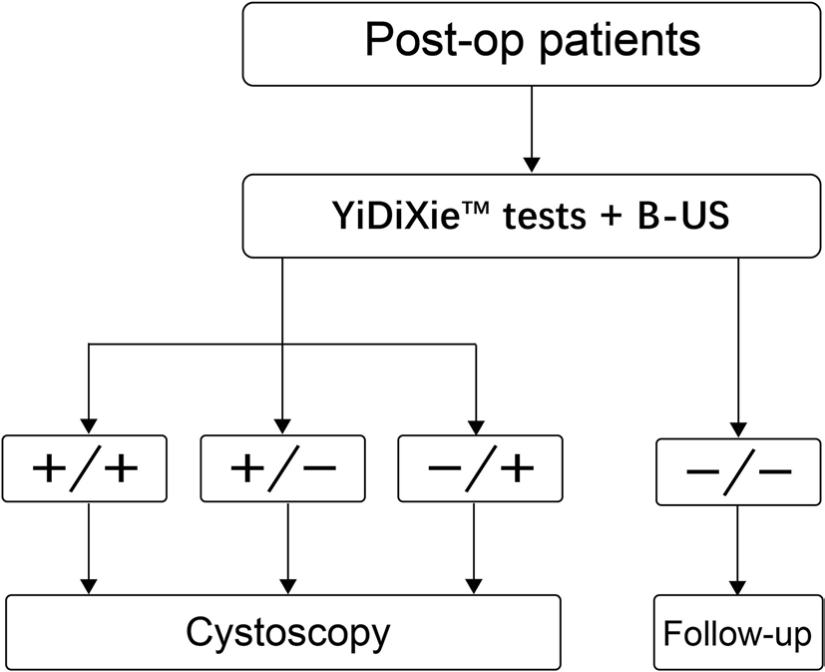
Recommended clinical pathway for YiDiXie™ tests in monitoring recurrence of uroepithelial cancer

The negative likelihood ratios of the combination of YiDiXie™ tests with B-ultrasound were all less than 0.1 (Tables 4, 5, and 6), so all three YiDiXie™ tests can effectively monitor the recurrence of urothelial carcinoma after surgery and are a possible alternative to cystoscopy. Clinicians can take corresponding intervention measures based on the recommended clinical pathways (Fig. 5), and the sensitivities and specificities of the three YiDiXie™ tests in combination with B-ultrasound vary. Clinicians can choose any one of the YiDiXie™ tests in combination with B-ultrasound based on the patient's needs and the local supply of cystoscopy resources.

The YiDiXie™ tests have significant advantages, mainly reflected in the low sample demand and operational convenience. This technology only requires a minimum of 20 μL of serum for testing, and the samples can be collected by oneself using a finger blood collection needle, without the need for venous blood collection. In addition, the testing process does not require professional medical personnel and equipment, and patients can collect the samples themselves and send them for testing. This model breaks through the limitations of traditional medical resources and provides a more convenient solution for tumor diagnosis.

This study has certain limitations. First, the number of cases in this study is relatively small. Future studies with larger sample sizes are needed to further evaluate. Second, the cases included in this study were patients with urothelial carcinoma and benign tumors of the urinary tract who were hospitalized or healthy individuals undergoing postoperative physical examinations. Future studies need to include cohort studies of natural populations before urothelial carcinoma treatment and after surgery to further evaluate. Finally, this study was a single-center study, which may lead to a certain degree of bias in the results. Multi-center studies are needed to further evaluate.

## Conclusion

In conclusion, YiDiXie™ tests have demonstrated considerable effectiveness in diagnosing urothelial carcinoma, and they have distinct levels of sensitivity and specificity. YiDiXie™-30 has a relatively high sensitivity and extremely high specificity in urothelial carcinoma. YiDiXie™-32 has a high sensitivity and high specificity in urothelial carcinoma. YiDiXie™-48 has an extremely high sensitivity and a relatively high specificity in urothelial carcinoma. YiDiXie™ tests have significant diagnostic value in urothelial carcinoma, improving the diagnosis of upper uroepithelial tumors and potentially providing an alternative to cystoscopy with CT or ultrasound.

## Data Availability

The datasets used and/or analyzed during the current study are available from the corresponding author on reasonable request.
